# Adenoma of the ampulla of Vater: a case report

**DOI:** 10.1186/1752-1947-8-228

**Published:** 2014-06-25

**Authors:** Konstantinos Alexiou, Argyrios Ioannidis, Ioannis Drikos, Athanasios Fotopoulos, Ioannis Karanikas, Nikolaos Economou

**Affiliations:** 1 Department of Surgery, Sismanoglion General Hospital, Sismanoglou 1, P.O. Box 15126, Athens, Greece

**Keywords:** Adenoma of ampulla of Vater, Transduodenal excision

## Abstract

**Introduction:**

Lesions of the ampulla of Vater are rare histological entities with an incidence of between 0.1 and 0.2% of gastrointestinal tumors. Until recently the main response to this kind of lesion was duodenopancreatectomy, regardless of the cellular atypia and local edema. In this study, we propose the application of transduodenal local excision of the ampulla of Vater especially in recognized cases of nonmalignant adenomas.

**Case presentation:**

In this case report we analyze the case of a 78-year-old Greek man who revealed symptoms such as icterus, abdominal pain without constipation and bloody stools. A physical examination showed painless swelling of the gallbladder (Courvoisier sign). No previous abdominal operations or hernias were identified. Blood tests, computed tomography scan analysis, gastroscopy and endoscopic retrograde cholangiopancreatography along with biopsies and cytological tests diagnosed nonmalignant adenoma of the ampulla of Vater with high-grade dysplasia. The treatment we followed was transduodenal local excision of his ampulla of Vater.

**Conclusions:**

Transduodenal local excision of the ampulla of Vater has limited side effects and postoperative complications, suggesting this particular technique to be the proper treatment for nonmalignant cases of adenomas.

## Introduction

Adenoma of the ampulla of Vater is a difficult-to-diagnose cause of obstruction of the biliary duct. It is among the rarest of gastrointestinal neoplasms. It is usually identified after gastroscopy followed by biopsies but often gets misdiagnosed. It carries a significant rate of morbidity and mortality.

## Case presentation

In this study we present a case of a 78-year-old Greek man who was diagnosed with adenoma of the ampulla of Vater. He presented in the emergency room with symptoms of abdominal pain without constipation and bloody stools. A physical examination showed icterus and Courvoisier sign.

**Figure 1:**
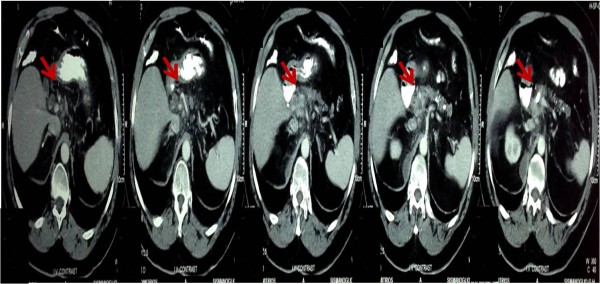
The abdominal computed tomography showed a mass at the ampulla of Vater (arrows).

The laboratory findings were: total bilirubin, 2.63mg/dL (normal 0.3 to 1.5); direct bilirubin, 1.33mg/dL (normal 0.01 to 0.35); indirect bilirubin, 1.50mg/dL (normal 0.01 to 0.35); serum albumin, 3.14g/dL (normal 3.5 to 5.0); alanine aminotransferase, 24U/L (normal 0 to 50); aspartate aminotransferase, 34U/L (normal 0 to 40); c-glutamyl transpeptidase, 27U/L (normal 0 to 40); alkaline phosphatase, 136IU/L (normal 30 to 140); C-reactive protein 221mg/dL (normal 0 to 10); white blood cell 8300/μL (normal 4000 to 10000); creatinine kinase 191U/L (24 to 195IU/L); carbohydrate antigen 19-9, 50.2U/mL (normal 0 to 37); cancer antigen 15-3, 45.2U/mL (normal 0 to 30).Ultrasonography of his abdomen revealed cholelithiasis and dilated intrahepatic and outer hepatic bile vessels. A computed tomography (CT) scan analysis showed a mass in his ampulla of Vater and pancreas, and revealed multiple calcified elements compatible with chronic pancreatitis (Figure 1). Lithiasis of his bile duct was also present. Magnetic resonance imaging (MRI) could not be done because of his pacemaker.

**Figure 2:**
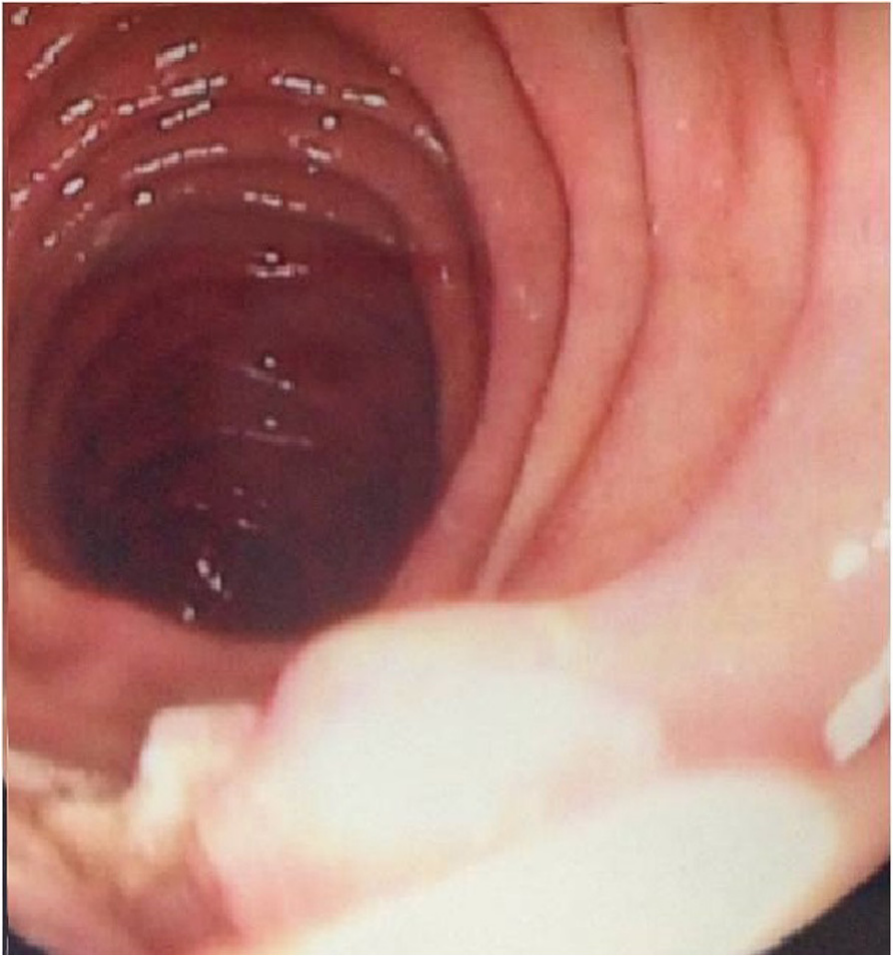
Gastroduodenal endoscopy showed a swollen duodenal adenoma of the ampulla of Vater with white mucosal covering.

Five days later endoscopic retrograde cholangiopancreatography (ERCP) was conducted. Entrance through the ampulla of Vater was impossible but a biopsy was taken for cytological examination. The histological analysis showed nonmalignant disease.Two days later common biopsies were taken during a gastroscopy (Figure 2) process and the results revealed nonmalignant papillary adenoma with high-grade dysplasia.

**Figure 3:**
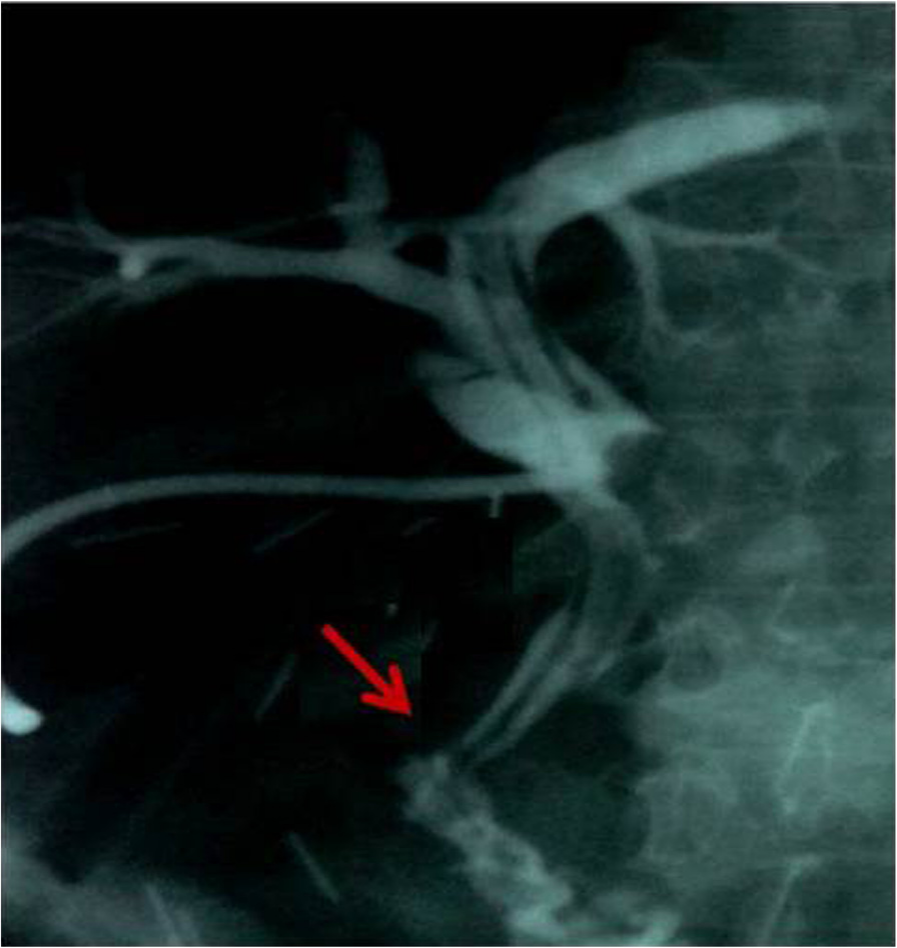
**The postoperative cholangiography reveals common bile and pancreatic duct able to drain into the duodenum.** The arrow indicates the pancreatic duct.

**Figure 4:**
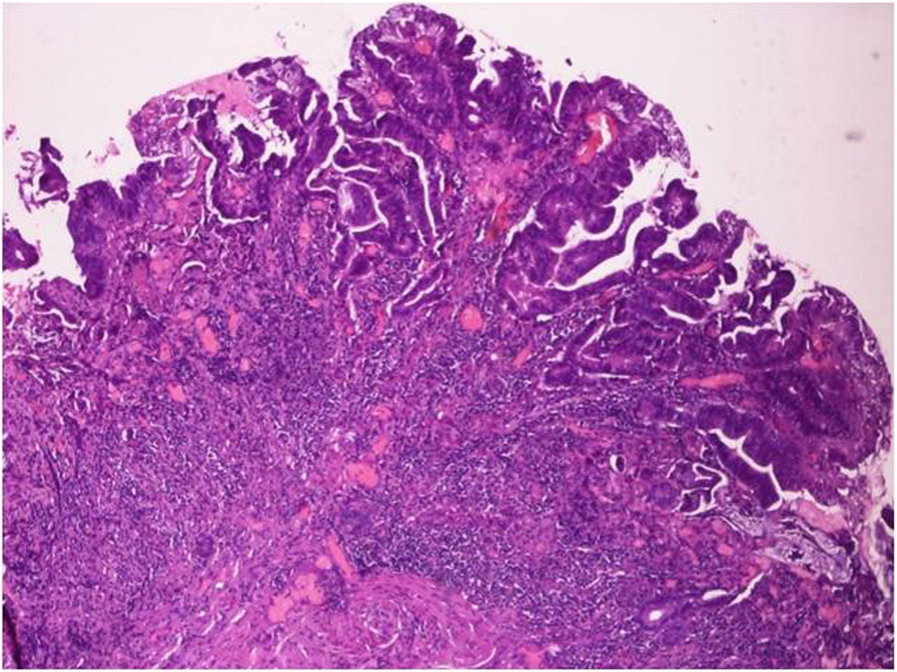
The histological analysis of the sample of the ampulla of Vater reveals adenoma with high-grade dysplasia and nonspecific inflammation with local corrosions.

Considering the size, the position and absence of malignancy we decided to proceed to transduodenal local excision of the adenoma. The operation consisted of transduodenal local excision of the adenoma of the ampulla of Vater, sphincterotomy of Oddi, followed by Oddi sphincteroplasty, exploration of his common bile duct and gastrointestinal anastomosis. Finally a Kehr tube was placed in his common bile duct through the incision of the exploration. The gastrointestinal anastomosis was conducted to reduce the inner pressure at his duodenum. The length of the procedure was 2 hours and 13 minutes and the estimated blood loss was approximately 280mL. Transfusion was not necessary as the his postoperative hematocrit was 35%.One week later a postoperative cholangiography (Figure 3) revealed no obstructions of his biliary tree and the results of his biochemical blood tests were normal.The final histological examination verified the biopsies from the gastroscopy. More specifically, the excision specimen consisted of mucosal and submucosal regions of his duodenum; it showed papillary adenoma with high-grade dysplasia, nonspecific inflammation and local corrosions, without evidence of malignancy (Figure 4).

He was discharged after a total of 16 days in our hospital.

## Discussion

Lesions of the ampulla of Vater are rare histological entities that in most cases have a high potential for malignancy [1]. Until recently, the main response to this kind of lesion was duodenopancreatectomy, regardless of the histological type, malignancy and local edema. However, improvements in diagnosis and therapy demonstrate the proper operation to be endoscopic removal of the adenoma when the tumor is detected. When endoscopic treatment is not possible, ampullectomy is an important technique compared with duodenopancreatectomy [1,2].

Adenoma of the ampulla of Vater was first described in 1895 by Calzavara [3] and diagnosis was difficult until 1973 when it became possible to implement ERCP in diagnosis of the biliary tree and pancreatic malformations. Adenomas of the ampulla of Vater are rare neoplasms that occur sporadically or in the context of genetic syndromes such as familial adenomatous polyposis and sometimes turn into malignant adenocarcinomas [4,5]. In terms of histopathological classification of neoplasmatic lesions of the ampulla of Vater, 40% are tubulovillous adenomas, 30% villous adenomas, 10% tubular adenomas and 20% are nonepithelial lesions such as endocrine adenomas or neurinomas. In addition, neoplasmatic lesions of the ampulla of Vater were found to range from 0.063 to 0.21% [6,7].

Tumors of the ampulla of Vater are usually identified by gastroscopy or ERCP. ERCP and endoscopic ultrasonography provide important information for the assessment of adenomas. With these techniques we can properly assess the intraductal extension of the tumor, demonstrating the best possible staging of the tumor toward the CT, the MRI and transabdominal ultrasound [8]. With the implementation of the evaluation of ERCP, the evaluation of adenomas is more accurate and intraduodenal surgical excision has become the proper treatment.

Furthermore, once adenomas of the ampulla of Vater have been identified they may be treated by surgical excision [8,9] and in patients with advanced size and cellular atypia, the surgical resection of adenoma seems to be the proper treatment option [10]. Although many patients reveal side effects after curative resection [11], most cases exhibit limited complications. The main complications include pancreatitis, postoperative bleeding and biliary tree inflammation [11].

Apart from intraduodenal resection of the ampulla of Vater, a pancreaticoduodenectomy may be used for the resection. This technique was first implemented in 1935 by Whipple *et al*. [12] and applied in a patient with carcinoma of the ampulla of Vater. In this study, the patient died after 25 months because of liver metastasis. This technique may be applied in cases of papillary adenocarcinoma and high-grade dysplasia. By contrast, Hoyuela *et al*. [13] reported a research study with four patients with adenomas of the ampulla of Vater who had a Whipple operation and showed no evidence of disease recurrence. The major advantages of the Whipple operation are the reduced risk of recurrence and the exclusion of sporadic adenomas presented in the ampulla of Vater. However, the technique has significant drawbacks mainly increased postoperative mortality and morbidity.

A research study conducting pancreaticoduodenectomies for Vater adenomas showed a mortality rate of up to 10% and morbidity rate from 25% to 65% [14]. In recent years the morbidity and mortality rates have improved but the longer hospitalizations have increased the cost of hospital care and possibly change quality of life [14,15].

In addition, patients with aggressive carcinoma of the ampulla of Vater who were treated with local excision, showed a high rate of recurrence after application of transduodenal local excision and a low rate of recurrence after Whipple surgery [15,16], suggesting that the application of transduodenal local excision is recommended when benign tumors are detected by histological analysis, as in our case.

Scientific data have already shown that the mortality rate (<1%) and the hospitalization time are lower after local ablation of adenomas of the ampulla of Vater. The advantages are also the lack of conducting a laparotomy and the necessity of general anesthesia [16-18], with the proviso that the patient be subject to regular postoperative monitoring in order to determine the risk of recurrence. Side effects such as pancreatitis and bleeding after transduodenal local excision seem to be self-limited and usually affected no more than 10% of patients. The temporary drainage of the pancreatic duct by using stents such as in our case may reduce postoperative acute pancreatitis.

## Conclusions

Adenoma of the ampulla of Vater is a rare nonmalignant tumor that cannot be diagnosed easily and carries significant rates of mortality and morbidity. Our therapeutic approach was selected in order to avoid postoperative complications such as those that might occur with duodenopancreatectomy. Even if endoscopic ampullectomy has been tried in some progressive institutions, further research to compare those two methods needs to be carried out in order to identify the proper therapy in cases of nonmalignant adenomas of the ampulla of Vater.

## Consent

Written informed consent was obtained from the patient for publication of this case report and any accompanying images. A copy of the written consent is available for review by the Editor-in-Chief of this journal.

## Abbreviations

CT: Computed tomography; ERCP: Endoscopic retrograde cholangiopancreatography; MRI: Magnetic resonance imaging.

## Competing interests

The authors declare that they have no competing interests.

## Authors’ contributions

KA, AI, ID, AF, IK, and NE carried out and participated at the surgical excision and the manuscript demonstration. KA, AI, and ID participated in the design of the study and helped to draft the manuscript. All authors read and approved the final manuscript.
